# Supplementary material for: MMP14 cleaves PTH1R in the chondrocyte-derived osteoblast lineage, curbing signaling intensity for proper bone anabolism
Source: eLife. 2023 Mar 9;12:e82142. doi: 10.7554/eLife.82142 (PMC10036123; doi:10.7554/eLife.82142)
Supplement: Figure 3—source data 1. [file elife-82142-fig3-data1.zip › Figure3-source data1.pptx]

## Slide 1
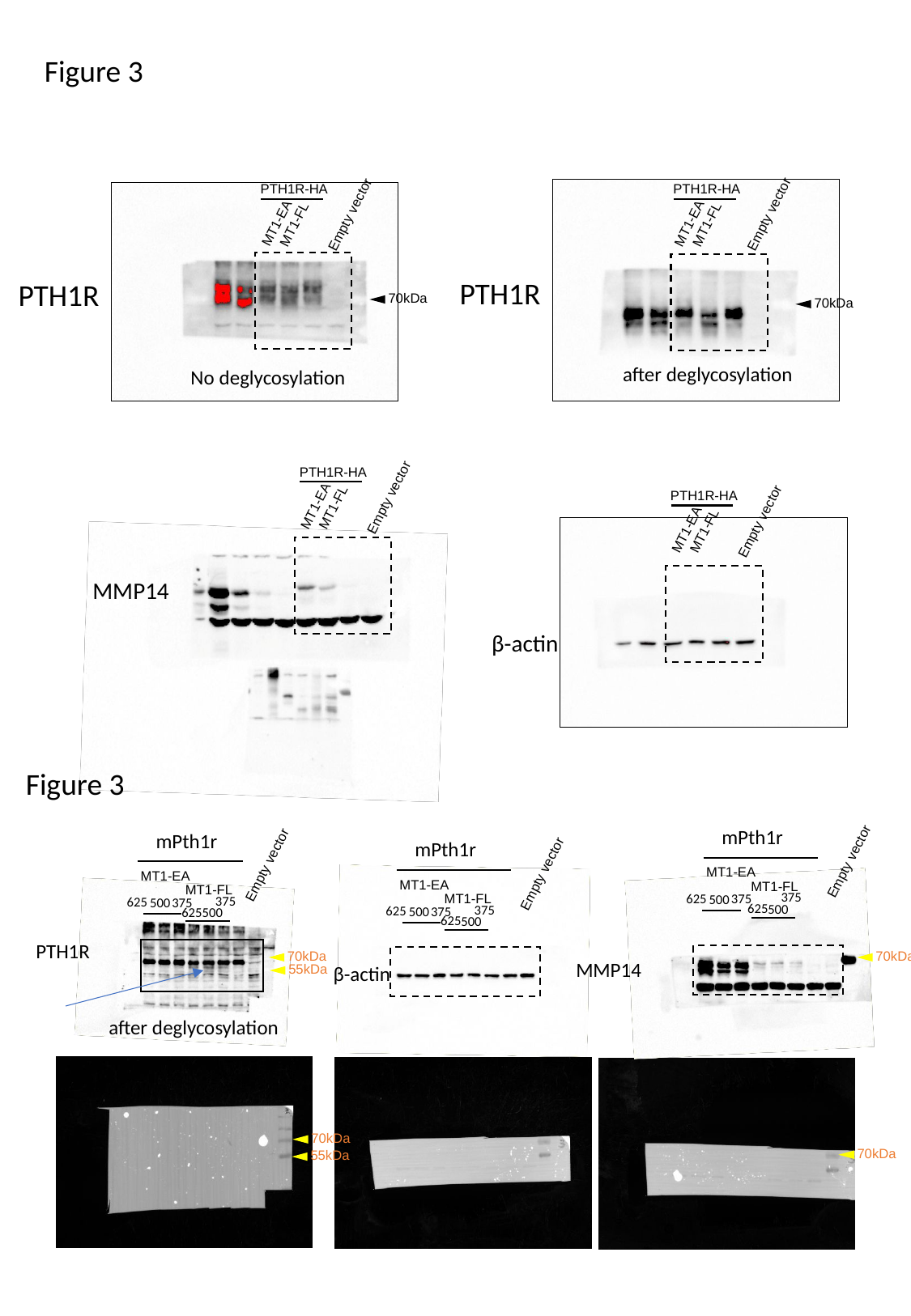

Figure 3
PTH1R-HA
PTH1R-HA
Empty vector
Empty vector
MT1-EA
MT1-EA
MT1-FL
MT1-FL
PTH1R
PTH1R
70kDa
70kDa
 after deglycosylation
No deglycosylation
PTH1R-HA
Empty vector
PTH1R-HA
MT1-EA
MT1-FL
Empty vector
MT1-EA
MT1-FL
MMP14
β-actin
Figure 3
mPth1r
mPth1r
mPth1r
Empty vector
Empty vector
Empty vector
MT1-EA
MT1-EA
MT1-EA
MT1-FL
MT1-FL
375
625
MT1-FL
375
500
375
625
375
500
625
500
375
625
375
500
625
500
625
500
PTH1R
70kDa
70kDa
MMP14
β-actin
55kDa
 after deglycosylation
70kDa
70kDa
55kDa

## Slide 2
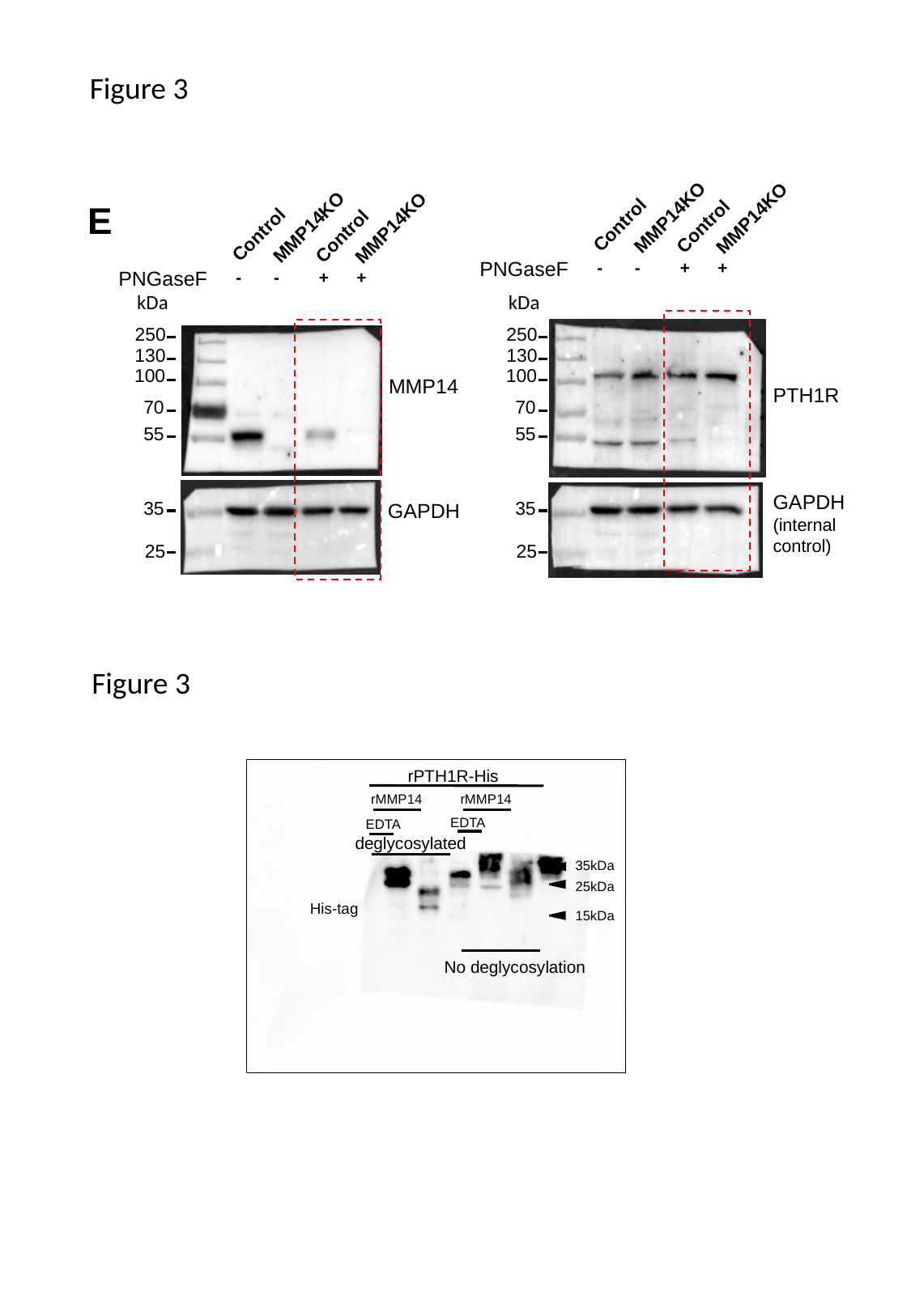

Figure 3
E
MMP14KO
MMP14KO
Control
Control
MMP14KO
MMP14KO
Control
Control
PNGaseF
-
-
+
+
PNGaseF
-
-
+
+
kDa
kDa
250
250
130
130
100
100
MMP14
PTH1R
70
70
55
55
GAPDH
(internal control)
35
35
GAPDH
25
25
Figure 3
rPTH1R-His
rMMP14
rMMP14
EDTA
EDTA
deglycosylated
35kDa
25kDa
His-tag
15kDa
No deglycosylation

## Slide 3
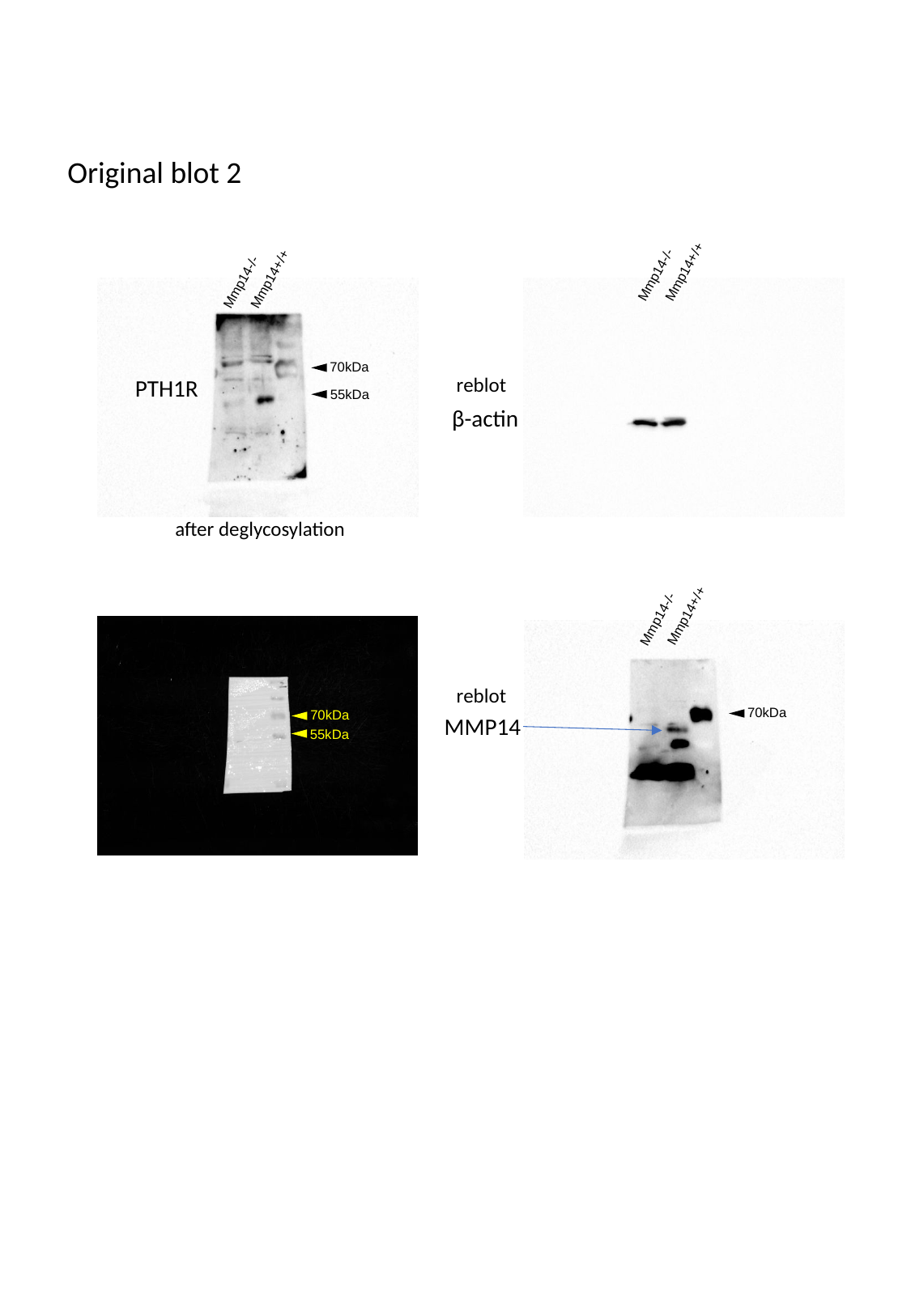

Original blot 2
Mmp14+/+
Mmp14-/-
Mmp14+/+
Mmp14-/-
70kDa
 reblot
PTH1R
55kDa
β-actin
 after deglycosylation
Mmp14+/+
Mmp14-/-
 reblot
70kDa
70kDa
MMP14
55kDa

## Slide 4
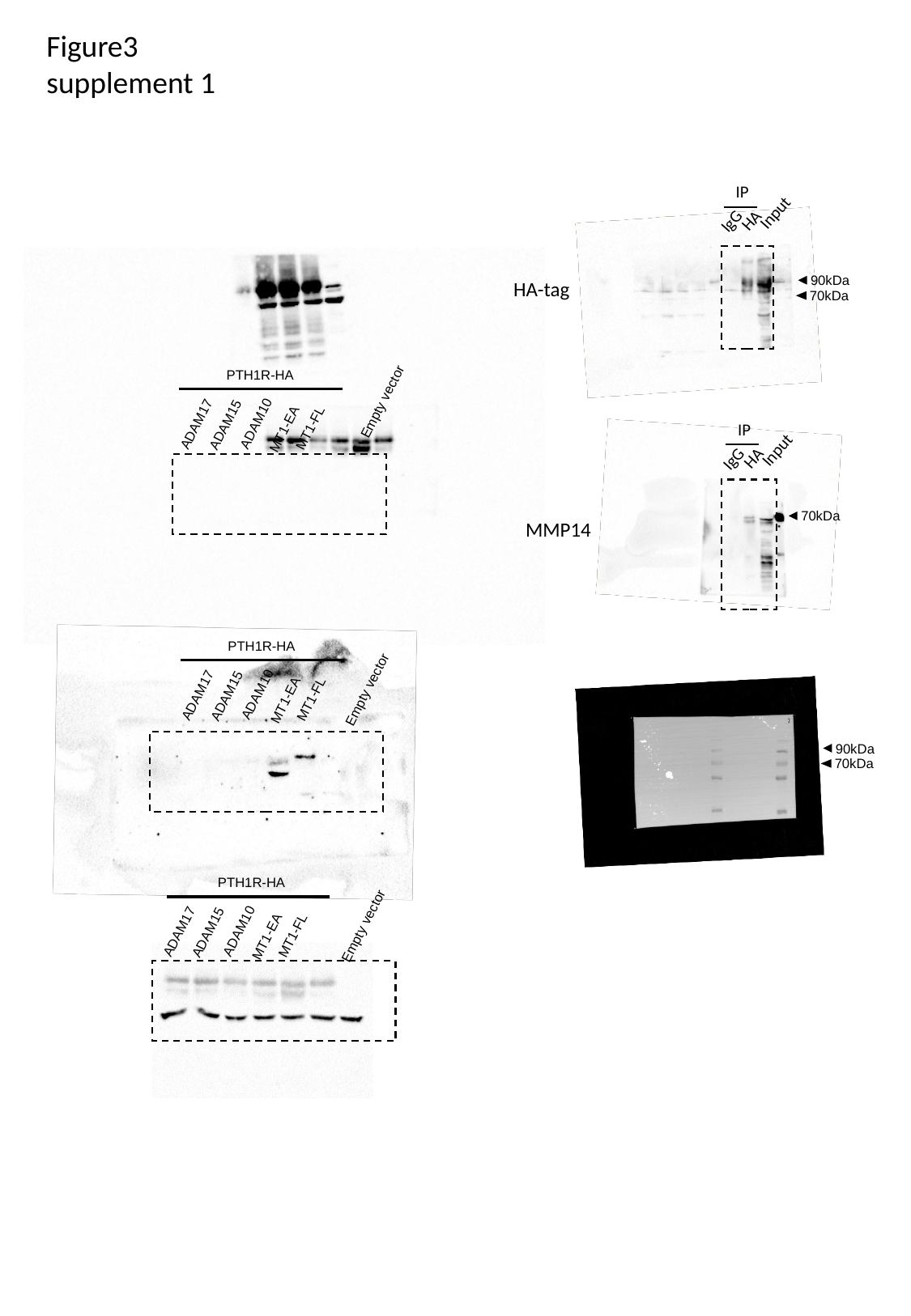

Figure3 supplement 1
IP
Input
HA
IgG
90kDa
HA-tag
70kDa
PTH1R-HA
Empty vector
ADAM10
ADAM17
ADAM15
MT1-EA
MT1-FL
IP
Input
HA
IgG
70kDa
MMP14
PTH1R-HA
Empty vector
ADAM10
ADAM17
ADAM15
MT1-EA
MT1-FL
90kDa
70kDa
PTH1R-HA
Empty vector
ADAM10
ADAM17
ADAM15
MT1-EA
MT1-FL

## Slide 5
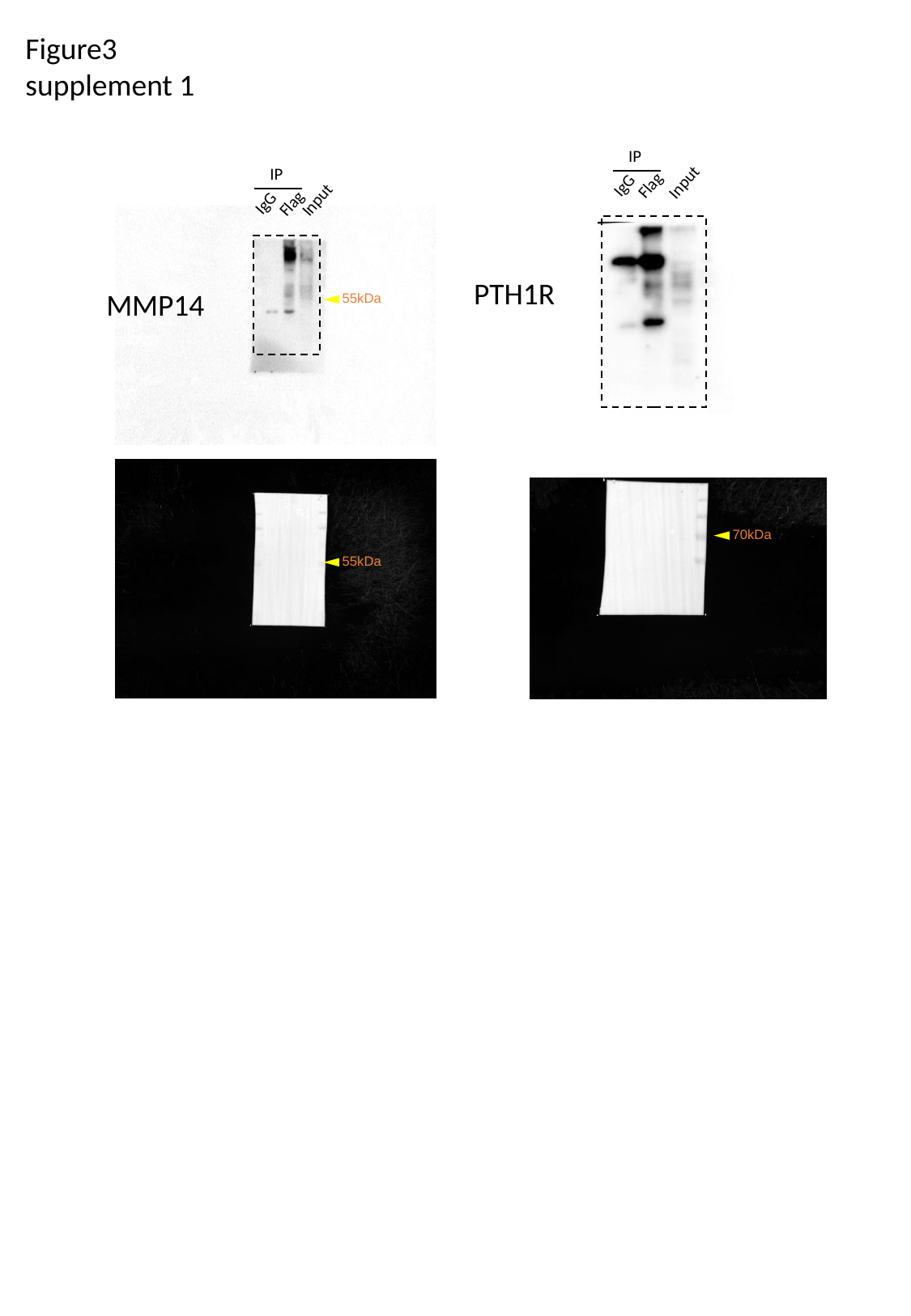

Figure3 supplement 1
IP
IgG
Flag
Input
IP
IgG
Flag
Input
PTH1R
MMP14
55kDa
70kDa
55kDa
