# Supplementary material for: MMP14 cleaves PTH1R in the chondrocyte-derived osteoblast lineage, curbing signaling intensity for proper bone anabolism
Source: eLife. 2023 Mar 9;12:e82142. doi: 10.7554/eLife.82142 (PMC10036123; doi:10.7554/eLife.82142)
Supplement: Figure 4—source data 1. [file elife-82142-fig4-data1.zip › Figure4-source data1.pptx]

## Slide 1
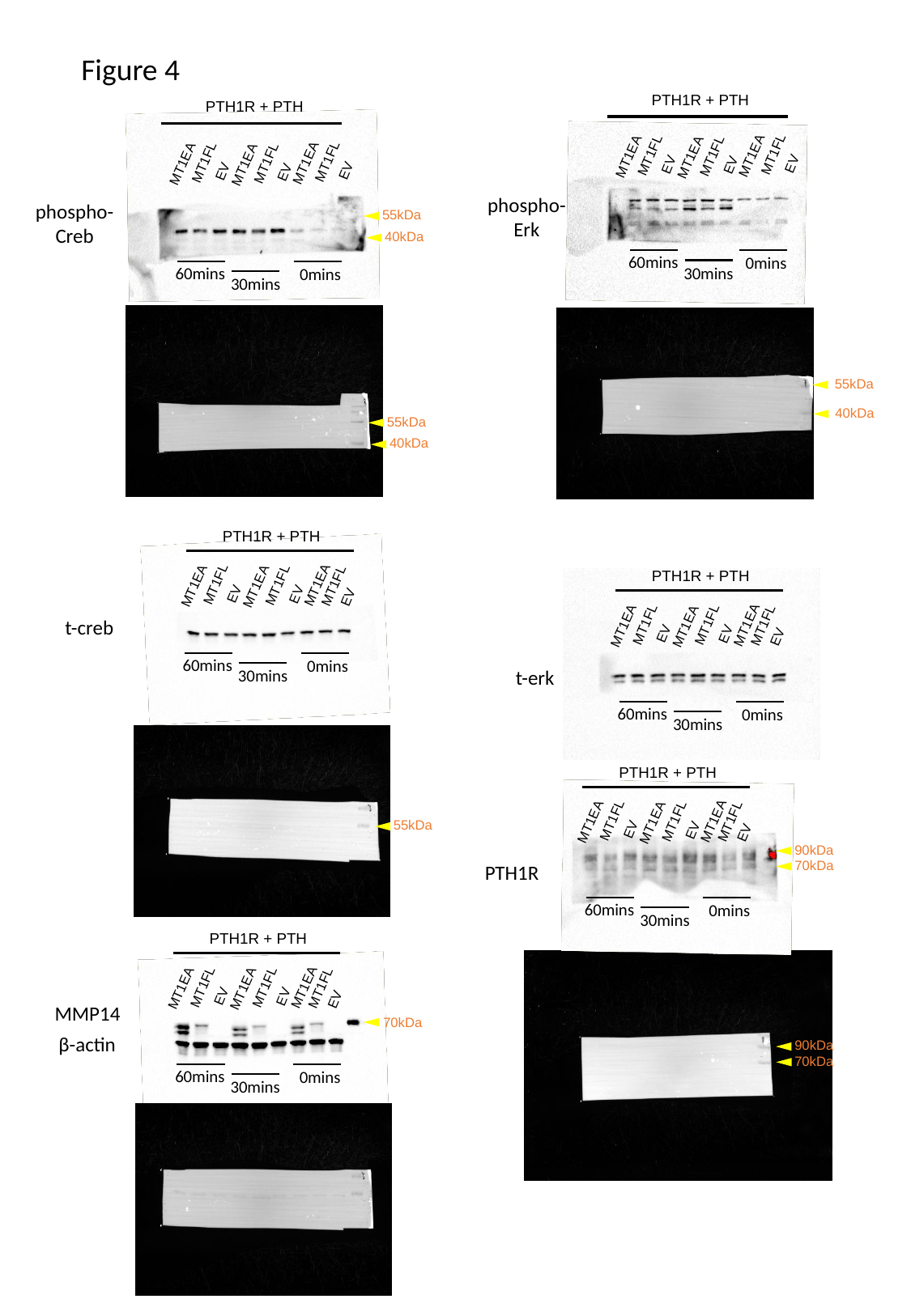

Figure 4
PTH1R + PTH
PTH1R + PTH
MT1FL
MT1FL
MT1FL
MT1EA
MT1EA
MT1EA
EV
MT1FL
EV
EV
MT1FL
MT1FL
MT1EA
MT1EA
MT1EA
EV
EV
EV
phospho-
Erk
phospho-
Creb
55kDa
40kDa
60mins
0mins
30mins
60mins
0mins
30mins
55kDa
40kDa
55kDa
40kDa
PTH1R + PTH
PTH1R + PTH
MT1FL
MT1FL
MT1EA
MT1FL
MT1EA
MT1EA
EV
EV
EV
MT1FL
MT1FL
t-creb
MT1EA
MT1FL
MT1EA
MT1EA
EV
EV
EV
60mins
0mins
t-erk
30mins
60mins
0mins
30mins
PTH1R + PTH
MT1FL
MT1FL
MT1EA
MT1FL
MT1EA
MT1EA
EV
55kDa
EV
EV
90kDa
70kDa
PTH1R
60mins
0mins
30mins
PTH1R + PTH
MT1FL
MT1FL
MT1EA
MT1FL
MT1EA
MT1EA
EV
EV
EV
MMP14
70kDa
β-actin
90kDa
70kDa
60mins
0mins
30mins

## Slide 2
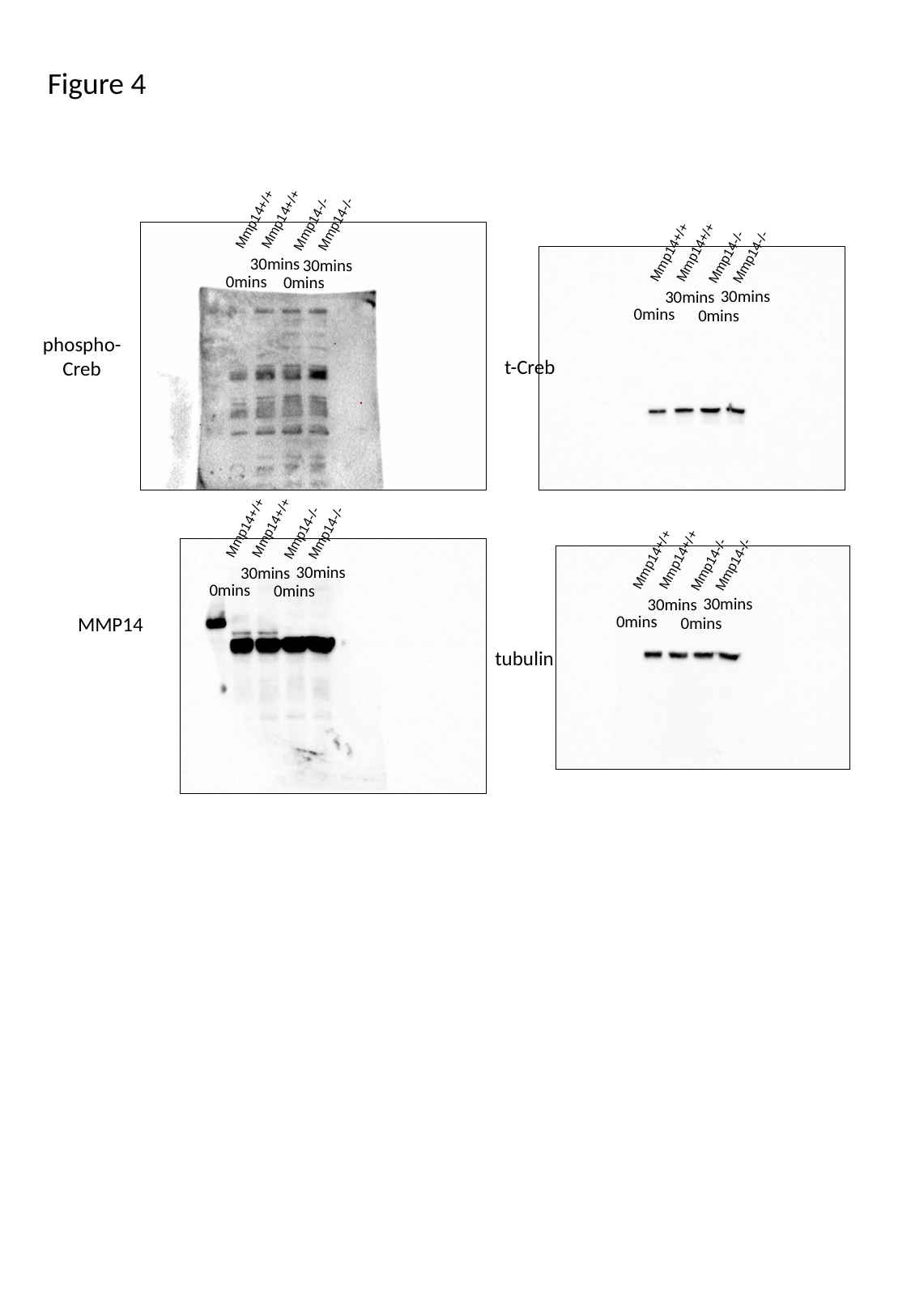

Figure 4
Mmp14+/+
Mmp14+/+
Mmp14-/-
Mmp14-/-
Mmp14+/+
Mmp14+/+
Mmp14-/-
Mmp14-/-
30mins
30mins
0mins
0mins
30mins
30mins
0mins
0mins
phospho-
Creb
t-Creb
Mmp14+/+
Mmp14+/+
Mmp14-/-
Mmp14-/-
Mmp14+/+
Mmp14+/+
Mmp14-/-
Mmp14-/-
30mins
30mins
0mins
0mins
30mins
30mins
0mins
MMP14
0mins
tubulin

## Slide 3
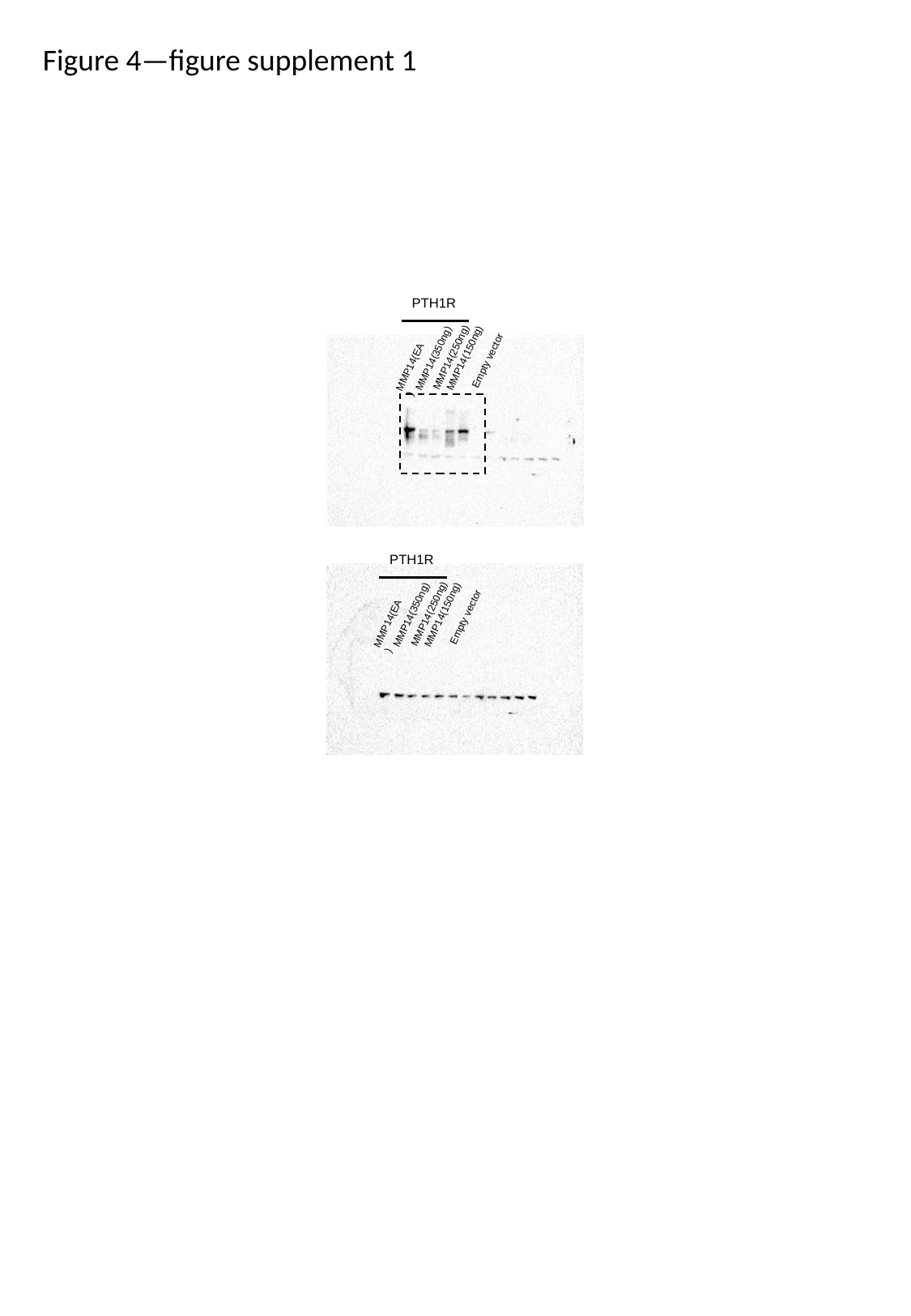

Figure 4—figure supplement 1
PTH1R
Empty vector
MMP14(250ng)
MMP14(350ng)
MMP14(150ng)
MMP14(EA)
PTH1R
Empty vector
MMP14(250ng)
MMP14(350ng)
MMP14(150ng)
MMP14(EA)
