# Supplementary material for: MMP14 cleaves PTH1R in the chondrocyte-derived osteoblast lineage, curbing signaling intensity for proper bone anabolism
Source: eLife. 2023 Mar 9;12:e82142. doi: 10.7554/eLife.82142 (PMC10036123; doi:10.7554/eLife.82142)
Supplement: Supplementary file 1. [file elife-82142-supp1.pdf]

List of Antibodies Used in this Manuscript

Antibody	Source	Catalogue number	ratio
**HA-tag**	Immunoway	YM3003	1:5000
**MT1-MMP (MMP14)**	Abcam	ab51074	1:5000 for WB<br>1:200 for IHC
**RFP**	SCIGEN		1:500 for IHC
**RFP**	Abcam	ab123754	1:200
**GFP**	ThermoFisher	A10262	1:1000
**Osterix**	Abcam	ab22552	1:200
**p-creb(S133)**	Abcam	ab43096	1:200
**t-creb**	Abcam	ab32515	1:1000
**p-erk**	Cell Signaling	CS4370	1:2000
**t-erk**	Cell Signaling	CS	1:1000
**PTH1R**	ThermoFisher	MA5-15676	1:1000
**PTH1R**	ThermoFisher	PA3-205	1:5000
**His-tag**	Immunoway	YM3004	1:1000
**β-actin**	Sigma		

List of ISH probes used in this Manuscript

Gene	Plasmid name	Enzyme	Description
Col1a1	pSp65-1	HindIII/Sp6(AS)	This plasmid contains a 0.8 kb gene fragment of the mouse *Col1a1* gene
Col2a1	pNJ61	HindIII/T7(S)<br>EcoRI/T3(AS)	This plasmid contains a 0.5kb cDNA fragment from exon 1 to 5 of mouse *Col2a1* gene
Col10a1	pRK26	EcoRI/Sp6(AS)<br>HindIII/T7(S)	This plasmid contains a 0.35kb cDNA fragment of exon 3 and 3' untranslated region from mouse *Col10a1* gene
Mmp14	pMT1E4	EcoRI/Sp6(AS)<br>HindIII/T7(S)	This plasmid contains a 300bps fragment from Exon 4 of *Mmp14* gene
Mmp13	M collase	HindIII/T7(AS)	731 bps derived from mouse *Mmp13* AvrII-HindIII fragment
Sox9	pSox9.5a	BamHI/T3(S)<br>HindIII/T7(AS)	This plasmid contains a 0.5kb DNA from 3' to the mouse *Sox9* gene.
Opn	mOP	EcoRI/SP6(AS)	This probe was derived from mouse Osteopontin mRNA fragment(157-1144)
